# Factors influencing men’s involvement in antenatal care services: a cross-sectional study in a low resource setting, Central Tanzania

**DOI:** 10.1186/s12978-019-0721-x

**Published:** 2019-05-09

**Authors:** Nyasiro S. Gibore, Theodora A. L. Bali, Stephen M. Kibusi

**Affiliations:** 1 Department of Public Health, School of Nursing and Public Health, College of Health Sciences, University of Dodoma, P.O. Box 395, Dodoma, Tanzania; 2 Department of Education, Faculty of Humanities and Education, Saint John’s University of Tanzania, P.O. Box, 47, Dodoma, Tanzania

**Keywords:** Male involvement, Men and antenatal care, Spousal pregnancy-related support

## Abstract

**Background:**

Men’s involvement can impact the delays in the decision to seek health care and in reaching a health facility, which are contributing causes for increased maternal mortality. Despite of the call to involve men in antenatal care, their participation is not well understood. This study aimed to determine the level of men’s involvement in antenatal care and the factors influencing their involvement in these services.

**Methods:**

A cross sectional study of 966 randomly selected men aged 18 years or older was conducted in Dodoma Region, from June 2014 to November 2015. Face to face interviews were conducted using a pretested structured questionnaire. The outcome variable was men’s involvement and was constructed from four dichotomized items which were scored zero to two for low involvement and three to four for high involvement. A multiple logistic model was used to measure the factors influencing men’s involvement in antenatal care services.

**Results:**

The level of men’s involvement in antenatal care was high (53.9%). Majority 89% of respondents made joint decisions on seeking antenatal care. More than half (63.4%) of respondents accompanied their partners to the antenatal clinic at least once. Less than a quarter (23.5%) of men was able to discuss issues related to pregnancy with their partner’s health care providers. About 77.3% of respondents provided physical support to their partners during the antenatal period. Factors influencing men’s involvement in antenatal care were occupation (AOR = 0.692, 95% CI = 0.511–0.936), ethnicity (AOR = 1.495, 95% CI = 1.066–2.097), religion (AOR = 1.826, 95% CI = 1.245–2.677), waiting time (AOR = 1.444, 95% CI = 1.094–1.906), information regarding men’s involvement in antenatal care (AOR = 3.077, 95% CI = 2.076–4.562) and men’s perception about theattitude of health care providers (AOR = 1.548, 95%CI = 1.090–2.199).

**Conclusion:**

Overall, more than half of respondents reported high involvement in antenatal care services. Access to information on men’s involvement, religion, occupation, ethnicity, waiting time and men’s perception about the attitude of care providers were significant factors influencing men’s involvement in antenatal care services in this study. Health promotion is needed to empower men with essential information for meaningful involvement in antenatal care services.

## Plain English summary

This study was carried out to determine the extent to which male partners participate in the care of their spouses during pregnancy and the factors influencing their participation. Data were collected from 966 men through face-to-face interview using a structured questionnaire. The study found that the majority (89%) of men made decisions on seeking pregnancy care with their partners. More than half (63.4%) of men accompanied their partners to seek pregnancy care at the health care facilityat least once. Less than a quarter (23.5%) of men were able to discuss maternal health issues related to pregnancy with their partner’s health care providers and the majority (77.3%)of men reduced the workload to their partners during pregnancy. Occupation, ethnicity, religion, waiting time at antenatal health care services, having information regarding men’s participation in pregnancy care and men’s perception about the attitude of health care providers toward men who accompanied their partners to pregnancy care services were the factors influencing male participation in care of their partners during pregnancy. Therefore, increasing community education and sensitization as well as creating couple-friendly reproductive health services could increase male participation in pregnancy care.

## Introduction

Antenatal care (ANC) is important for the health of the mother and the development of foetus because it links the woman and her family with the health care system which may increase the chance of using a skilled attendant atbirth and contributes to good health through the life cycle [1]. Men’s involvement in ANC has potential to reduce delays in decisions to utilize antenatal health care services. In African countries, men’s involvement in ANC has been shown to increase utilization of maternal health services [2–6]. Before the 1994 International Conference on Population and Development in Cairo, reproductive health programmes were focused on women’s health viewing men as non-actors whose role was irrelevant [7]. Recently, however, there has been increasing attention on the role of male participation in women’s reproductive health after recognizing that men’s attitudes, knowledge, and behavior can strongly influence women’s health choices [8–12].

Despite the call for men’s involvement in maternal care, men are rarelyinvolved in their partners’ care during pregnancy [13]. The main reason for this lack of involvement is that reproductive health has largely been viewed as a women’s concern [14–16]. Recent scholars have raised the point that many men may not be prepared to participate in antenatal care [17] and that ANCsettings create barriersfor male involvement [18–22]. Inadequate male involvement in ANC can lead to persistent increases in maternal morbidity and mortality [23], because men hold the decision making power on where and when women should seek health care services, particularly in African settings [5, 10, 24].

Sub-Saharan Africa has the highest maternal mortality ratio of about 510 maternal deaths per 100,000 live births [25].In Tanzania, 2014 estimates indicated7,900 maternal deaths and a maternal mortality ratio (MMR) of 410 per 100,000 live births, which is a decline by 55% between 1990 and 2013 [25]. Although Tanzania has madeprogress in reducing MMR, the rate was far from the target of the fifth Millennium Development Goal of reducing MMR to 230 per 100,000 live births by 2015 [26]. As the Millennium Development Goals have now changed to Sustainable Development Goals [27], more efforts are needed to achieve the target of reducing MMR to less than 70 per 100,000 live births by 2030 [28].

Causes of maternal deaths have been well studied [29]. In Africa, South of Sahara these causes can be closely linked with low female socio-economic status as well as lack of decision making opportunity among women about their health care and over household budget [30]. Men’s involvement can potentially impact the first two delays in maternal care. First, the delay in making a decision to seek health care which may be caused by under-estimation of the severity of the problem and the need for male partners’ approval to seek care, commonly reported among women in developing countries [24, 31, 32]. The delay in reaching a health care facility may be associated with a lack of money for transport as well as other health care related costs in which women depend on a male partner. An earlier report showed that 42% of women in Dodoma Region reported that lack of money for transport was a barrier for timely access of health care services [31].

Awareness among men on pregnancy related problemsand their complicationsis very low [33], this may consequently limit their scope of involvement in maternal care. In Tanzania, men rarely help their wives/partners in infant care and household chores during the maternity period and those who do are culturally portrayed as being effeminate and weak [34]. Little has been documented in Tanzania, particularly in Dodoma Region regarding the level of men’s involvement in ANC. This study aimed at assessing the level and the factors influencing men’s involvement in maternity care during antenatal period in Dodoma Region.

## Methods and material

### Study settings and design

The study was conducted in Dodoma Region, Central Tanzania. Dodoma Region is located in central part of Tanzania, covering an area of 41,310 km^2^, with a population of 2,083,588 people and population density of 50 people per square kilometers [35]. The region’s health care service structure is made up of seven hospitals, 32 health centers and 269 dispensaries, most of which provide antenatal care services. The area was selected because it is a lower resource region and represents cultural aspects of male dominance and women dis-empowerment and that limited studies have addressed this phenomenon to date. It was assumed that studying the level of men’s involvement in ANC in Dodoma Regionwould provide a broad picture of the study findings from different cultures in Tanzania.The region has seven Districts: Bahi, Chamwino, Chemba, Kondoa, Kongwa and Mpwawa districts as well as Dodoma Municipality. Four Districts namely, Kondoa, Kongwa, Chamwino and Dodoma Municipality were purposively selected as regional representative of the distinctive characteristics of each district regarding male involvement.

The study design was a quantitative cross-sectional survey. The study involved married men aged 18 years and above, who had children aged two years or below at the child’s last birthday. The men must have resided with their spouse together in the same household and their partners must have had a second or more pregnancy at the time of data collection. The data were collected from June 2014 to November 2015.

### Sample size and selection

#### Sample size estimation

The sample size was obtained using the Kish Leslie’s formula [36] as shown below.

$$ \mathrm{N}=\frac{t^2\times p\left(1-p\right)\mathrm{D}}{m^2}. $$

Where by:

**N ═** required sample size, **t ═** confidence level at 95% (standard value of 1.96), **p ═** estimated prevalence of men attendance at ANC which was 46% from previous study [37], **m ═** margin of error at 5% (standard value of 0.05) and **D** = design effect (assumed to be 2)

$$ \mathrm{N}=\frac{(1.96)^2\times 0.46\left(1-0.46\right)2}{(0.05)^2} $$

$$ \mathrm{N}=764 $$

The minimum sample size was 764 respondents. The sample was further increased by 10% to account for contingencies such as non-response or recording error resulting to a total sample of 841.

#### Selection of study participants

Respondents were recruited into the study through a household survey. A multistage sampling technique was used for selecting the sample units. In the first step, the four districts werepurposively selected out of seven districts. In the second step, two wards (sub divisions) were randomly chosenfrom each district using a table of random numbers to make a total of eight wards. In the third step, one village or street (in case of municipality) were chosen randomly from each of the eight wards using a table of random numbers, making a total of six villages and two streets namely; Mnenia and Pahi (Kondoa), Mlanga and Makang’wa (Kongwa), Mlowabarabarani and Chinangali-II (Chamwino) and Msalatoand Nzuguni (streets) (Dodoma Municipality). In each selected villages and streets, 105 eligible households were chosen to participate in the study which makes a total of 840 households. Systematic sampling technique with the starting point obtained using a table of random number was used to select the houses in each village or street. The sampling interval of three was used to pick the house. The first house to be interviewed was randomly selected by randomly pointing in the random number table while eyes closed so as to obtain the starting number. From the first house, every third house was selected till sample size was obtained. The direction of movement was determined by random selection. In the household if a man had more than one partner with a child born within the past two years, the interview was conducted based on the information from the youngest child.

### Data collection

A pre-tested, structured, interviewer-administered questionnaire was used to collect the data. The questionnaire was developed by the researchers, it consisted of both open and close ended questions and it was divided into two parts. The first part captured information on household social demographic variables. The second part assessed the level of men’s involvement in ANC. The questionnaire was prepared in English and translated into Swahili. To ensure accuracy in the translation, the questionnaire was back translated into English by two independent nursing officers who were familiar with ANC in Dodoma. Pre-testing of the tool which involved 100 men was carried out at Ng’ong’hona village which is located in Makulu Ward in Dodoma Municipality. The questionnaire was administered by eight trained research assistants who were community development workers from the four Districts involved in the study.

### Measurement of variables

The dependent variable (men’s involvement in ANC) was constructed as a single variable to obtain the involvement index by using four dichotomized (yes/no) variables namely: 1) accompanying partner to antenatal care services, 2) providing physical support during antenatal period, 3) joint planning of when and where to seek antenatal care,and 4) discussingmaternal health issues with health care providers during antenatal period. The variable physical support was measured by asking the respondents the following question: - How did you share household work with your partner compared to the times when she is not pregnant? The responses were as follows; (1) the same work (2) more than usual and (3) not at all. Those who answered options (1, 2) were regarded as having provided physical support to their partners while option (3) was regarded as no physical support to their partners. The responses were coded as “yes” for physical support and “no” for no physical support. To obtain the level of men’s involvement in ANC by using the above mentioned four variables, each variable scored one if performed and zero if not performed. A total score was calculated by adding the score of each activity reported to be performed by a respondent. The level of involvement was classified as follows: a score of zero to two was regarded as a low level of involvement, a score of three to four as high level of involvement. Previous studies applied this approach of categorization [38, 39].

### Independent variables

Attitudes were measured by asking the respondentsthe following question: How do you find the attitude of health workers towards men who accompany their wives to hospital to seek care? The question had two options:1) They attend to us very well and friendly and 2) They are unfriendly. Those who answered option one had a positive attitude and number two were regarded as having a negative attitude.

### Data analysis

Data were entered and analyzed by using Statistical Package for Social Sciences (SPSS Version 21.0). Univariate analysis was performed to obtain frequency and percentage for the demographic variables and the level of involvement in different activities of antenatal care. *Chi-square* test was performed to draw out possible associations between the men’s involvement and background characteristics. Bivariate and multivariatelogistic regression analysis was carried out to determine the factors influencing men’s involvement in antenatal care. The significance level was set at a *p*-value of < 0.05.

## Results

### Socio-demographic characteristics of respondents

Although the estimated sample size for this study was 841 respondents, during conducting of the survey, a total of the 966 married men participated in the study. This was a response rate of 100%, which suggests the interest of respondents in the topic. A higher response rate helps the researcher to be sure that the findings are representative and promote confidence in the results [40]. It also helps in controlling unforeseeable confounders. The socio-demographic characteristics of the study participants are shown in Table 1. The age of respondents ranged from 18 to 70 years. Over 62%of the respondents were between 25 and 44 years. The number of children per respondent ranged from one to twenty-two children. Over 67%of respondents had one to four children. The majority (74.1%) of the respondents was Christian and most (74.5%) belonged to the Gogo and Rangi ethnic groups. About 70.4% of the respondents were peasants (cultivators and agro pastoralist). The majority of respondents (77.5%) had a primary education, and 14.1% never attended any formal schooling. A total of 91.5% of respondents were currently in monogamous relationships while the remaining were in polygamous relationships. Most, of the respondents (94.2%), reported being in a marriage in which they chose their partner, while the remaining had their marriage arranged by family or relatives. The majority of respondents (85%) reported to live less than five kilometers from a health facility.

**Table 1:** Socio-demographic characteristics of respondents (*N* = 966)

Variable	Category	N (%)
Age	15–24	56 (5.8)
	25–34	294 (30.4)
	35–44	309 (32.0)
	≥ 45	307 (31.8)
Occupation	Employed	80 (8.3)
	Agricultural	653 (67.3)
	Agro pastoral	30 (3.1)
	Business	144 (14.9)
	Casual labourer	59 (6.1)
Education level	No education	136 (14.1)
	Primary education	749 (77.5)
	Secondary education	62 (6.4)
	Tertiary education	19 (2.0)
Ethnicity	Gogo	515 (53.3)
	Rangi	205 (21.2)
	Sandawe	4 (0.4)
	Kaguru	86 (8.9)
	Hehe	21 (2.2)
	Others (Fipa, Mbulu, Ngoni)	135 (14.0)
Religion	Christian	716 (74.1)
	Muslim	250 (25.9)
Marriage relationship	Arranged marriage	56 (5.8)
	Chosen each other	910 (94.2)
Marriage type	Monogamous	884 (91.5)
	Polygamous	82 (9.5)
Number of children	1–2	327 (33.9)
	3–4	322 (33.3)
	≥ 5	317 (32.8)
Distance to health facility	≤ 5 km	854 (85.0)
	≥ 5 km	110 (15.0)

### Level of men’s involvement in antenatal care

**Fig. 1:**
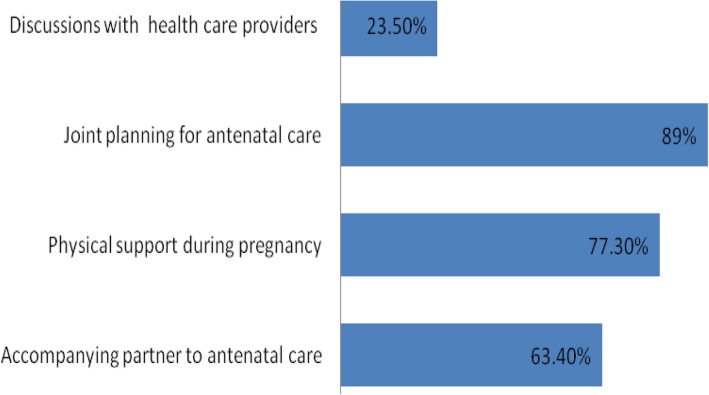
Level of Men’s Involvement in Antenatal Care

The level of men’s involvement in ANC was assessed by using the variables shown in Fig. 1 below. Findings show that 46.1% of respondents had a low level of involvement, while 53.9% had high level of involvement. The majority of respondents (89%) made joint decisions on seeking antenatal care. The majority of respondents (77.3%) reported that they provided support such as workload relief to their partners during the antenatal period. More than half of respondents (63.4%) accompanied their partners to the antenatal clinic at least once. However, less than a quarter of men (23.5%) was able to discuss pregnancy-related health issues with their partner’s health care providers.

Table 2 below, presents the association between the background characteristics of respondents with the level of men’s involvement in ANC. Belonging to Gogo ethnic group was associated with high level of involvement in ANC (61.6%) compared to belonging to other ethnic groups (45.2%,*p* < 0.001). Christian menhad a higher level of involvement (58.7%) compared to Muslim men (40.6%) (p < 0.001). There was little difference in the level of involvement between marriage types (monogamous vs polygamous) as well as between arranged or forced marriage, these differenceswerenot statistically significant. Men who reported to spend less than one hour to receive health services had a higher level of involvement (57.9%) compared to men who reported to spend more than one hour (51.6%) to receive health care services (*p* < 0.05). There was an association between access to information on men’s involvement in ANC and the level of men’s involvement in ANC (*p* < 0.001). Low level of involvement in ANC was more prevalent (65.9%) among men with poor access to ANC information (p < 0.001).

**Table 2:** Association between Background Characteristics of Respondents and Level of Men’s Involvement in ANC (*N* = 965)

Variables	Level of involvement		Total	χ2	*P*-value
	Low involvement	High involvement			
	n(%)	n(%)	n(%)		
Age					
15–30	100 (44.8)	123 (55.2)	223 (100)		
> 30	345 (46.4)	398 (53.6)	743 (100)	0.175	0.676
Occupation					
Employed	141 (50.9)	136 (49.1)	277 (100)		
Not employed	304 (44.1)	385 (56.0)	689 (100)	3.656	0.056
Education level					
No education	37 (51.4)	35 (48.6)	72 (100)		
Primary education	343 (45.7)	408 (54.3)	751 (100)		
Above primary education	65 (45.5)	78 (54.5)	143 (100)	0.889	0.641
Ethnicity					
Gogo	198 (38.4)	317 (61.6)	515 (100)		
Others	247 (54.8)	204 (45.2)	451 (100)	25.777	***
Religion					
Christian	296 (41.3)	420 (58.7)	716 (100)		
Muslim	149 (59.6)	101 (40.6)	250 (100)	24.865	***
Marriage relationship					
Arranged Marriage	415 (46.5)	477 (53.5)	892 (100)		
Chosen each other	30 (40.5)	44 (59.5)	74 (100)	0.985	0.321
Marriage type					
Monogamous	26 (46.4)	30 (53.6)	56 (100)		
Polygamous	419 (46.0)	491 (54,0)	910 (100)	0.003	0.955
Time					
≤ 1 h	149 (42.1)	205 (57.9)	354 (100)		
> 1 h	296 (48.4)	316 (51.6)	612 (100)	3.555	*
Access ANC information					
Yes	354 (42.8)	474 (57.2)	828 (100)		
No	91 (65.9)	47 (34.1)	138 (100)	25.599	***
Number of children					
1–4	306 (47.7)	335 (52.3)	641 (100)		
≥ 5	139 (42.8)	186 (57.2)	325 (100)	2.143	0.143
Distance to health facility					
≤ 5 km	405 (47.4)	450 (52.6)	855 (100)		
≥ 5 km	40 (36.0)	71 (64.0)	111 (100)	5.078	*
Attitude of health care providers					
Positive	353 (44.2)	445 (55.8)	798 (100)	6.189	*
Negative	92 (54.8)	76 (45.2)	168 (100)		

*Statistically significant at *p-value< 0.05, **p-value< 0.01, ***p-value< 0.001

Men who reported to be living more than five kilometres from a health care facility had a higher level of involvement in ANC (64%), compared to their counterpart (52.6%) (*p* < 0.05). Men’s perception about the attitude of health care providers towards male involvement in maternal care was associated with high level of men’s involvement in ANC (p < 0.05). Age, occupation, education and number of children were not associated with the level of men’s involvement in ANC.

Table 3, shows the multivariate logistic regression analysis for the factors influencing men’s involvement in ANC. Variables which showed a significant relationship with male involvement after *chi-square* analysis were added in a multivariate model to estimate their independent association. Thereby, controlling for confounding variables including occupation, ethnicity, religion, waiting time, access to information, number of children, distance to the health care facility and perceived attitude of care providers. The variables which remained statistically significant were: occupation, ethnicity, religion, time spent to receive antenatal care services, access to information regarding men’s involvement in ANCand perceived attitude of care providers. Having employment decreased the chances of men’s involvement in ANC (AOR = 0.692, 95%CI = 0.511–0.936, *p* < 0.05). Similarly men’s perception about the attitude of health care providers toward men who accompanied their partners at ANC decreased the chances of men’s involvement in ANC (AOR = 0.568, 95%CI = 0.400–0.808,*p* < 0.01). Men from Gogo ethnic group were more likely to be involved in ANC than men from other ethnic groups (AOR = 1.495, 95%CI = 1.066–2.097, *p* < 0.05). Christians were more likely to be involved in ANC compared to Muslim (AOR = 1.826, 95%CI = 1.245–2.677, p < 0.01). Men who reported to spend less than one hour to receive health services were more likely to be involved in ANC as compared to their counterparts (AOR = 1.444, 95%CI = 1.094–1.906, p < 0.05). Likewise, men who reported to have heard information regarding their involvement in ANC were three times more likely to be involved in ANC, compared to their counterparts (AOR = 3.077, 95%CI = 2.076–4.562, *p* < 0.001).

**Table 3:** Multivariate Logistic Regression Analysis of the Factors influencing High level Men’s Involvement in Antenatal Care

Variable	Crude OR (95% CI)	P-value	Adjusted OR (95% CI)	*P*-value
Occupation				
Employed	0.739 (0.541–1.009)	0.057	0.692 (0.511–0.936)	*
Not employed	1			
Ethnicity				
Gogo	1.507 (1.071–2.121)	*	1.495 (1.066–2.097)	*
Others	1			
Religion				
Christian	1.799 (1.225–2.643)	**	1.826 (1.245–2.677)	**
Muslim	1			
Time				
≤ 1 h	1.480 (1.118–1.959)	**	1.444 (1.094–1.906)	*
> 1 h	1			
Access to Information				
Yes	3.027 (2.039–4.494)	***	3.077 (2.076–4.562)	***
No	1			
Number of children				
1–4	0.801 (0.600–1.757)	1.071		
≥ 5	1			
Distance to health facility				
≤ 5 km	0.717 (0.465–1.104)	0.131		
≥ 5 km	1			
Attitude of health care providers				
Positive	1.548 (1.090–2.199)	*	0.568 (0.400–1.808)	
Negative	1			

*Statistically significant at *p-value < 0.05, **p-value < 0.01, ***p-value< 0.001

## Discussion

Generally, more than half (53.9%) of men had high level of involvement in ANC. The level of involvement in this study is higher than findings from other studies [6, 41]. The difference observed could be due to a difference in methods employed to construct the involvement level as well as study setting. For example, previous studies were hospital based while the current study was community based. The high level of men’s involvement in ANC in this study implies the effectiveness in implementing safe motherhood initiatives which emphasizes on male involvement in the region [29]. The initiative integrates men’s involvement in maternal health particularly in individual birth preparedness, and prevention of mother to child transmission of HIV [29]. The multivariate analysis revealed that exposure to information regarding men’s involvement in ANC was the strongest factor influencing men’s involvement in ANC, as it has been described in other studies [37, 41–43]. It is more likely that, exposure to ANC information has a great potential in addressing misconceptions and myths that hinders men from being involved in maternal care. Other scholars suggested that men who know the danger signs of pregnancy are more likely to act fast to save the lives of their wives when complications arise [44].

By and large, more than half (63.4%) of the men reported to accompany their partners, at least once, to an antenatal clinic visit. This is consistent with previous studies in Uganda and Nigeria where the proportion of male involvement in ANC was 65.4 and 63% respectively [45, 46]. Shared cultural values on gender roles among African societies could explain the observed similarities in these findings [14–16].In this study more than half of respondents who accompanied their partners to an ANC visit reported to spend more than one hour in health care facility waiting for services. This is likely to discourage men from coming to ANC in subsequent visits. Studies done in other parts of Africa show that the longer the time spent waiting for services, the less the chances are for men to be involved in ANC services [4, 47, 48]. Time spent in accompanying spouses to ANC services could have more implications to male involvement in ANC among employed men. Findings from the current study showed that having employment was negatively associated with the level of men’s involvement in ANC. Men who are in the paid workforce, are often not in a position to spend virtually the entire day participating in ANC services [41].

Thus, health care providers and program implementers should take appropriate action to advocate and encourage men’s involvement in ANC. During ANC, pregnant women and their partners are given health education. This may result in a greater outcome on maternal health behaviors compared to when women receive this education alone [5]. It is understood that education and health services provided during the antenatal period have the potential to reduce pregnancy and delivery complications and improve birth outcomes [49]. Thus if men and women miss this opportunity during ANC, it is not surprising that Sustainable Development Goal number three is not achieved.

Surprisingly, our study has revealed a negative association between the perception of participants on the attitude of health care providers toward men who accompany their partners to ANC and the level of men’s involvement in ANC. Participants who perceived positive attitude of providers had low odds of involvement compared with those who had a negative perception. This finding was not expected but could be explained in terms of the protective nature of men towards their partners whereby those who had a negative perception may accompany their partners as a way of protecting them from providers with a negative attitude. However, further studies are needed to explore in depth the perception of men on provider’s attitude towards men’s involvement with ANC in settings where gender roles are still highly observed in caring for pregnant mothers.

This study found that the majority of men (89.0%) reported that they made joint decision with their partners regarding ANC. This finding differs significantly from previous studies where joint decision making within couples was reported as low as 9% in Nigeria () and 28.6% in India [50]. The reason for this variation could be due to the effect of cultural differences and limited exposure to safe motherhood initiative programs. In this study, 63% of men reported to have accompanied their partners to ANC visit at least once. Continued health education given to both women and men at RCH clinics coupled with the influence of safe motherhood initiatives including ongoing media campaigns such as the *WazaziNipendeni* Project may have further contributed to high male involvement which ultimately increased couple’s joint decision making on ANC issues. This could further explain the high proportion (77.3%) of men who reported to offer support to their partners including relieving them from some of the household chores during pregnancy. Previous studies have reported low proportion of men offering such support to their partners [7, 50–53]. It has been observed that involving partners in maternal health care and encouraging joint decision-making among couples may provide an important strategy in improving men’s involvement and couples empowerment [39]. This study therefore, stresses the need for health care providers, voluntary groups, religious and community leaders, to encourage inter-spousal communication during sensitization of the community on the importance of men’s involvement in ANC.

This study revealed that religion was an important factor for men’s involvement where men of the Christian faith reported relatively higher involvement in ANC than their Muslim counterparts. Studies conducted in Nigeria and Cameroon had similar findings [15, 47]. Future intervention should address religion as an important platform for implementation. Factors such as male user friendly ANC services should be emphasized, to address some of the religious values including having a separate space for men accompanying their spouses to ANC clinics [15, 47] to minimize risk of intermingling between clinic attending non-spousal men and women.

Providers need to be trained on culturally sensitive provision of care to men accompanying their spouses to ANC clinic. In this study only 23.5% of men accompanying their partners reported getting a chance to discuss maternal health issues with the providers. These findings imply that majority of men who accompanied their partners to ANC (63.4%) did not have contact with health care providers of their partners. Apart from infrastructural barriers including shortage of providers and limited space in the consultation rooms, providers may have limited competence to provide culturally sensitive care to accompanying male partners [37, 38, 41, 54].

This study provided some important information on men’s involvement from a large group of participants in four districts of the Dodoma Region. Therefore, it may be possible to generalize the findings to the whole region. However, there are a few limitations that need to be mentioned. The study assessed only four variables for men’s involvement in ANC, while the variable men’s involvement is complex. It needs to be assessed by a combination of several variables. The combination of other variables such as financial support for ANC, arranging transportation for delivery, planning for a potential blood donor, involvement in decision making of the location for delivery, accompaniment to the place of deliverycould improve the accuracy of the measure. Although the study included men with children aged less than two years, the issue of recall bias and the fact that pregnancy issues may not have as much importance to the male partners as they do to the female may limit the study findings. Additionally there are chances that, social desirability could have played a negative influence on men to disclose their involvement in maternal care issues especially given the cultural context of gender roles in the study area.

Future research should explore men’s expectations of ANC services and test ways to meaningfully integrate them in couple friendly ANC services. Also there is a need to assess the cultural competency of care providers in attending male partners accompanying their spouses for ANC services. Factors such as religion were observed to significantly influence men’s involvement in ANC. Therefore, there is a need for studies to explore potential barriers that are strongly connected to religious beliefs and see how religion could be effectively used as an avenue for testing health promotion interventions including appropriate media messages targeting men’s involvement in ANC and other reproductive health services.

## Conclusion

Overall, more than half of respondents reported high involvement in ANC services. Involvement was high in terms of accompanying partners to ANC, providing physical support during pregnancy and making joint decisions for ANC whereas discussing maternal health issues with the providers was very low. Access to men’s involvement information, religion, occupation, ethnicity, time spent waiting for ANC and men’s perception about the attitude of health care providers toward men who accompanied their partners to ANC were significant factors influencing men’s involvement in ANC services in this study. Health promotion is needed to empower men with essential information for meaningful involvement in ANC services. Future interventions should address among others; cultural competence of providers in involving men accompanying their spouses in the ANC service model as well as creating couple-friendly reproductive health services.

## Abbreviations

ANC: Antenatal care
AOR: Adjusted odd ratio
CI: Confidence interval
HIV: Human immune deficiency virus
MMR: Maternal mortality ratio
OR: Odd ratio
SPSS: Statistical Package for Social Sciences
